# New Researches on Acute and Articular Rheumatism, &c.

**Published:** 1838-10

**Authors:** 


### ART. VI.

*Nouvelles Recherches sur le Rheumatisme articulaire aigu en général, et spécialement sur la Loi de Coincidence de la Pericardite et de l'Endocardite avec cette Maladie, ainsi que sur l'Efficacité de la formule des Emissions sanguines coup sur coup dans son Traitement.* Par J. BOUILLAUD, Prof. de Clin. Med. à la Faculté de Méd. de Paris. —*Paris,* 1836. 8vo. pp. 159.

*New Researches on Acute and Articular Rheumatism in general, and especially on the Law of Coincidence of Pericarditis and Endocarditis with this Disease, as well as on the Efficacy of quickly repeated Bleedings in its Treatment.* By J. BOUILLAUD.—8vo. pp. 159. 1836.

*Leçons de Clinique Médicale faites à l'Hôtel-Dieu de Paris.* Par le Prof. A. F. CHOMEL: *recueillies et publiées par* A. P. REQUIN, D.M.P. Tome II. (*Rheumatisme et Goutte.*)—*Paris,* 1837. 8vo. pp. 524.

*Lectures on Clinical Medicine, delivered at the Hôtel-Dieu, Paris.* By Professor A. F. CHOMEL: *reported and published by* Dr. A. P. REQUIN. Vol. II. (*Rheumatism and Gout.*)—8vo. pp. 524. 1837.

THE distinction of any class of diseases as *specific* is very unsatisfactory. The word is but a threadbare cloak of ignorance: and, although it may be said that we actually *know* but little of any disease or class of diseases, still there are certain tolerably uniform characteristics which enable us to arrange some of them with apparent correctness. Take, for instance, those forms of inflammation of organs about which opinions are nearly uniform. It is in these cases that examination after death has, when connected with symptoms and signs of disease during life, afforded a ground of classification: but, in the disease which we are about to consider, together with a great variableness in symptoms, there is the most scanty pathological history; so that, whilst many of its phenomena oblige us to regard it as inflammatory, it is, as it were, excluded from inflammations by the term *specific.* It must be expected that, under these circumstances, medical authors will scarcely be likely to agree; and the works before us are a curious specimen of this difference of opinion. Two physicians, enjoying similar opportunities of observation, living in a city where it can scarcely be supposed but that the disease which they were occupied in investigating must have presented itself in a similar form to each, are directly at variance respecting the etiology, the pathology, the symptoms of some of its attendant lesions, and the treatment of the disease. M. Bouillaud's work was published the first: in it he maintains that, in the majority of the cases of acute rheumatism, there are certain inflammatory affections of the heart; claiming, at the same time, the merit of having made this discovery: he asserts the purely inflammatory nature of the disease; he places it in certain tissues of the joints, and vaunts the efficacy of large and repeated bleedings for its cure. Although the present work of M. Chomel was published subsequently to that of M. Bouillaud, most of his opinions on the disease had been previously stated in a thesis, published in 1813, (and which is reprinted at the end of this volume,) and in journals and other works. He denies the frequency of the cardiac affection, and all M. Bouillaud's merits as a discoverer; maintains that the disease is not essentially inflammatory; that its primitive seat is not that stated by M. Bouillaud; and that the treatment recommended is quite unjustifiable.

M. Bouillaud's pamphlet is written with great spirit, but is the result of much less experience than the work of M. Chomel. The former confines his investigations to acute articular rheumatism: the latter considers the disease in all its forms. M. Bouillaud laughs at the notion of essential or idiopathic fevers: his opponent admits them. They both enter into the defence of their own notions with very considerable bitterness, and they are both led beyond the strict limits of truth in the support of their respective opinions.

In the present article we shall take M. Chomel as our guide, and shall now proceed to give, at considerable length, an analysis of his work; and, in order that there may be no misapprehension of the author's views, we must state, *in limine*, that he considers gout and articular rheumatism to be the same disease. It is necessary for the reader to bear this in mind; not only to understand the author's own views, but to estimate justly the value of the facts and arguments which he opposes to the views of M. Bouillaud. In our analysis we shall follow the order adopted by M. Chomel, leaving, as he has done, to the last chapter, our opinions as to the correctness of his classification.

Rheumatism, says M. Chomel, constitutes a natural class of diseases, distinguished by, 1, *their seat in fibrous organs;* 2, *their mobility*, or the facility with which they pass from one part to another; 3, *their intermittence*,—i. e. more or less frequent and sudden alternations of disappearance and recurrence. To these may be added a fourth characteristic, the *diversity of their forms:* for example, though rheumatism of the joints most frequently appears with heat, redness, pain, and swelling, there may be only pain, generally increased by pressure. Morgagni has cited one case of change in muscular structure from rheumatism; but this does not appear (as we shall hereafter see) to be a case of any value. M. Chomel knows no other case, nor is he aware of any change produced by rheumatism in muscles: hence this rheumatism, often characterized by pain only, cannot be regarded as an inflammatory disease. It is rare, but such has been the case, that pain is alone met with in articular rheumatism. It has more than once happened that no appreciable lesion has been found in the joints of rheumatic patients who have suddenly died of some other affection. Here, then, are two opposite forms of rheumatism; one, with the accompaniment of inflammatory lesions; the other, without appreciable organic change in the dead body. In a third species, rheumatism affords examples of lesions which approximate to *organic lesions, properly so called;* swelling of the parts is added to the pains, together with the formation of tophaceous concretions, erosion of cartilages, &c. Lastly, in some cases of articular rheumatism, where the most marked phenomenon, next to the pain, is an effusion into the cavity of a joint, which is serous and perfectly limpid, the disease has the true form of *essential dropsy* of the ancients.

Anatomical examination does not discover the seat of rheumatism. It is only after the observation of symptoms that the muscles were regarded as its seat; and to what class of their fibres it is to be referred still remains a question. But, as fibrous tissues are attacked where there is no muscular tissue,—e. g. the tendo Achillis,—it is probable that the aponeurotic expansions covering muscles are the parts affected in muscular rheumatism. It is but a matter of presumption that the fibrous tissues of joints are the parts first affected. M. Bouillaud, however, regards the essential seat of articular rheumatism as the synovial membrane. It is a question difficult, if not impossible, to decide, from the absence of morbid changes after death, and from the circumstance that, when these are found to exist, various tissues are implicated; and the question remains as to which was first affected. We content ourselves, therefore, with expressing our authors' opinions. In internal rheumatisms, following the same analogy, M. Chomel considers that the fibrous tissues are the parts affected; such, for instance, as exist in the heart, stomach, &c.

The division adopted by M. Chomel, and which, in our remarks on each of the volumes which stand at the head of this article, we shall follow, is this:

*Order* 1. Muscular Rheumatism; or rheumatism of the voluntary muscles.

*Order* 2. Articular Rheumatism; or rheumatism of the joints.

*Order* 3. Visceral Rheumatism; or rheumatism of certain fibrous organs situated within the splanchnic cavities.

Order I. Muscular Rheumatism.—1. *Situation.* Every region of the body may be affected by it, but it is more frequent in the trunk than in the limbs. Lumbago, torticollis, and pleurodynia are the most common kinds; and, when the limbs are attacked, it is in those parts of them which are nearest the trunk. 2. *Etiology.* In many cases it is brought on by cold, fatigue, &c.; but very frequently it depends on that condition of the body called rheumatic diathesis, which will be discussed in another place. 3. *Symptoms.* The essential symptom is pain, increased by contraction of the muscle: this pain is not attended, as inflammatory pains are, by increased heat; on the contrary, a sensation of cold sometimes accompanies it; there is neither swelling nor redness, and generally no febrile symptoms. 4. *Mobility.* It is very subject to change from one muscle to another, sometimes extending to the next muscles, or to the corresponding muscle of the opposite side of the body. 5. *Complication with articular rheumatism.* Sometimes the muscles and joints are attacked at the same time; in other cases alternately. This coincidence has been remarked by all good observers, and it is of importance, as tending to prove the identity of the disease. It is not rare in acute, but it is frequent in chronic rheumatism. 6. *Termination.* Always by resolution. Some cases will be found in print which were supposed to be rheumatism terminating in suppuration; but we fully agree with our author that it is most probable, in these cases, that the diagnosis was imperfect, and that the formation of matter was in the origin overlooked, and the pain consequent upon it mistaken for, or attributed to, rheumatism.

1. *Muscular Rheumatism of the Head.* *a.* In the epicranial region. This may attack the whole or some part of the fibro-muscular layer covering the cranium: sometimes it is confined to the nucha, sometimes to the vertex, sometimes to the sinciput, sometimes to one half of the cranium, when it is called hemicrania. The motions of the occipitofrontalis muscle and pressure increase the pain, which in some cases is exasperated by everything producing a determination of blood to the head, so that some patients cannot cover their heads or bear the heat of a pillow.

The treatment recommended is, the application of leeches two or three times behind the ears and temples, and exposing the head or merely lightly covering it. If the complaint is obstinate and severe, the head should be partially shaved and leeches applied, followed by a blister: where the pain becomes chronic, it may be advisable to cover the head warmly, so as to encourage perspiration; for this purpose a cap of oiled silk is advisable. Our author gives some sage cautions as to the danger of such warmth in some cases producing apoplexy; and, where such a dangerous tendency exists, he recommends a contrary treatment, gradually lessening the coverings of the head until the patient can bear complete exposure. This fear of apoplexy we take to be very absurd, and calculated to make the young practitioner cautious in trying the most effectual remedy in hemicrania, keeping the part warm.

*b.* In other regions of the head. M. Chomel has sometimes observed rheumatism of the masseter, producing pain and difficulty in mastication. In some rheumatic patients, he has also seen the motions of the palpebræ become painful. The following case is one of rheumatism of the muscles of the eyes.

*Case.* Marie Rabure, æt. 28, servant, was admitted into the Hospital of la Charité, the 19th July, 1815. Both her parents were rheumatic. For some time previously she had been subject, for four or five days together, to pains of the knees or shoulders. Eight days before admission she had suffered from rheumatic pains of the nucha, preventing her turning her head from side to side; and a similar pain in the eyes, so that she could neither elevate nor depress them, nor turn them to either side, without suffering acutely. When she wished to look on one side, she turned her whole body. These symptoms remained for about three days, when she was attacked with typhoid fever, which proved fatal. On examination, no changes were detected either in the nucha or in the muscles of the eyes. Morgagni relates a case in which the tongue was affected with rheumatism, and a similar one has fallen under M. Chomel's observation. The muscles of the pharynx are sometimes affected with rheumatism.

2. *Rheumatism of the Muscles of the Neck, or "Torticollis."* This, which we vulgarly call "stiff neck," is generally produced by cold. The author recommends leeches, if the pain is very severe; in slighter cases flannel, or in obstinate ones blisters; and, if these do not relieve, he applies from a fourth to half a grain of the acetate or muriate of morphia on the blistered surface.

3. *Rheumatism of the Thoracic Muscles, or Pleurodynia.* This often occurs without any manifest cause; sometimes it is produced by cold, and sometimes by the exertion of coughing or sneezing. It is generally confined to a small space on one side of the chest, and very often (like the pain in pleurisy) near the nipple; but it may affect every part of the chest: sometimes one whole side is affected, and this guides the diagnosis; for it very seldom happens that pleurisy or pneumonia produces pain over so extended a surface. Dr. Gaudet, who has published a series of cases of pleurodynia, found that, in eleven out of thirteen cases, the pain attacked the left side. The importance of this observation could only be satisfactorily estimated by a much larger number of cases. The pain is very intense, much more so generally than in pleurisy; it frequently forces the patient to cry out, which is hardly ever the case in pulmonary diseases. The pain is increased by motion of the trunk, by inspiration, expiration, and by pressure. Lying on the painful side is more distressing than in pleurisy, and very often the pain is aggravated by moving the arm of the affected side. Add to these symptoms the absence of physical signs of pleurisy and pneumonia, the apyrexia, which is the rule in pleurodynia, and the want of cough, except in such cases as the cough is the cause of the disease. But, if pleurodynia continues, it is necessary to be watchful for the occurrence of pleurisy. Hence it is desirable to treat the disease actively from its commencement. Pleurodynia may be confounded with pains of the stomach, spleen, and liver, more easily than with inflammations of the pleura or lungs. A minute examination of the antecedent circumstances and existing symptoms is here necessary to clear the diagnosis. Emollient applications will often suffice to remove a pleurodynia. A hot omelette, and a loaf just taken from the oven, are popular remedies in France. A large cupping-glass, the air of which is well rarified, has succeeded in removing the pain: but, if the pain be obstinate, local depletion, followed by one or more blisters, will be required. Pitch plasters are useful in obstinate but not very active forms.

4. *Rheumatism of the anterior and lateral Parietes of the Abdomen.* This has no particular name. When trifling in degree, it passes unnoticed; but, when violent, it may be mistaken for gastritis, enteritis, or peritonitis: and this accounts for its having been little noticed. The first symptoms presented by such a case are, indeed, very like those of peritonitis. The abdomen is so sensitive that the knees are kept elevated to prevent the bedclothes resting upon it: the most moderate pressure causes intolerable pain, and the expression of countenance is that of peritonitis. But, with these symptoms, the pulse is not excited; there is no fever, no nausea or vomiting; food and drink pass as usual, and the abdomen yields a normal sound to percussion. Dr. Gesnet has observed this rheumatism in puerperal women, and regards the state of the system existing in them as its frequent cause. Many of the cases called puerperal peritonitis he believes to be of this nature; and the violent efforts made by the abdominal muscles in aid of the uterine contractions are regarded as capable of accounting for it; as, in other muscles, forced exercise is an ordinary cause of rheumatism. To this state are referred those cases termed by Dr. Gooch "*nervous affection of the peritoneum.*"* One of the cases quoted by our author from Dr. Gooch died, and no morbid appearances were found. The cause of death in this instance is supposed to have been over-bleeding; and, indeed, such was Dr. Gooch's opinion. But, whether the cases above mentioned were of a rheumatic character, or affections of the peritoneum of a nervous character, it is perhaps difficult, without more information, to decide. In neither case

* See two cases at pages 63 and 67 of Gooch's "Account of some of the most important Diseases peculiar to Women."

would examination after death afford any satisfactory evidence; for muscular rheumatism appears to be characterized by no appreciable morbid appearances; and it was the absence of morbid changes which was the groundwork of Dr. Gooch's opinion. There are, however, some points of difference which prevent our agreeing entirely with our author on this point. It is said that the abdominal rheumatism is much more obstinate (in respect to treatment) than torticollis or pleurodynia: Dr. Gooch's cases yielded to opium and fomentations very speedily; in one case within twenty-four hours. In the rheumatic cases, it is said that, "together with the most alarming signs, there is no trouble in the pulse:" Dr. Gooch speaks of his cases as accompanied with "pain and tenderness of the abdomen, with a rapid pulse." The French author, laying down rules of treatment, says, after commenting on the "intrinsic tenacity of abdominal rheumatism, or rather the superaddition of peritoneal inflammation," "Be prodigal in (*prodiguez*) the use of leeches, until there is no formal contraindication:" Dr. Gooch speaks of his cases as "not requiring bleeding, as not relieved by it, but as speedily relieved by fomentations and opiates."

We do not, however, regard these circumstances as showing clearly that the two terms do not include the same disease. The mere quickness of pulse, and perhaps the effect of treatment in Gooch's cases, are not of much importance as distinguishing signs; because rheumatism is so varying in its character, and a woman's pulse is disposed to be quickened after labour. We mention them only as facts requiring further observation.

The sign, almost pathognomonic, of abdominal rheumatism is, that pressure, although very painful, does not produce so much pain as motion of the body: hence the patient lies constantly on his back. Peritonitis may be developed during the course of this rheumatism. The knowledge of this fact, and of the obstinacy of the disease itself, must influence the treatment. Repeated use of leeches will not always subdue the disease; but it is, happily, rare to find it resist proper treatment, and become chronic. The copious application of leeches, revulsives on the skin of every kind, baths (in which the patient should be allowed to remain a long while,—four, five, six, or even eight hours,) are the remedies to be employed.

5. *Rheumatism of the Lumbar Muscles: Lumbago.* Under this section we shall only notice the author's remarks upon a case related by Morgagni, of an individual, apparently affected by chronic lumbago, in whose body, after death, the lumbar muscles were found changed in colour and consistence. This appears to be a solitary fact of the supposed structural changes produced by rheumatism in muscles. A young goldsmith suffered from a pain in the right lumbar region, which yielded to no remedies. When this had lasted a year, the left side became similarly affected. The cervical region became also affected with pains, apparently rheumatic. Paralysis of motion of the inferior limbs followed, and tympanitis before death. The thick fleshy mass, which serves as a common origin to the sacro-lumbalis and longus dorsi muscles, was, for about five fingers' breadth, longitudinally and transversely, of the colour of old furniture made of walnut. This change of colour was not alone superficial, but affected the substance of the fleshy mass as well as the subjacent muscles. Throughout the whole of this discoloured mass, the muscular fibres were very loose and soft, separated in many places by effused and grumous blood. These lesions were the more apparent the nearer to the spine.*

Is this lesion, says M. Chomel, to be attributed to the rheumatic affection? and he answers the question negatively. Morgagni himself suspects, in order to explain the paralysis of the lower limbs, that a lesion analogous to that found in the muscles existed in the nervous branches constituting the crural plexus. The lumbar pain, doubtless, was connected with this lesion of the muscles: but it is denied that this lesion, which was not traced beyond the loins, and which probably extended to the spinal nerves, was an affection proper to rheumatism. We certainly think with M. Chomel, that from such a case, imperfectly examined as it was, nothing can with certainty be inferred as to the organic changes produced by rheumatism in muscular structures.

6. *Muscular Rheumatism of the Limbs.* This is the more frequent, the nearer to the trunk. It is very mobile, and has a great variety of seats. It springs, as it were, from one limb to another, and from one muscle to another in the same limb. The differential diagnosis of rheumatism appearing in these parts, it will be evident, is important when the various sympathies which exist between them and the other organs of the body and the painful affections hence produced are considered. But it would lead us too far to enter minutely into these. That, however, which distinguishes all these sympathetic pains from the idiopathic pains of rheumatism is, that the former oblige the patient constantly to change his place, in the vain hope of obtaining a more advantageous position; whilst absolute rest is observed by the rheumatic patient. Certain cases of neuralgia, the hysteric pains to which some individuals are subject, the pains in the bones accompanying syphilis, those which are produced by lead, and those which attend scurvy, are referred to, and distinguished from the characteristics of muscular rheumatism of the extremities. It is only necessary for us to mention them, to show in what the differential diagnosis must consist. The treatment of this is to be conducted on the same principles as that of the previous forms.

Order II. Articular Rheumatism. We will here again remind our readers that M. Chomel includes gout under the above head, believing, as he does, in the identity of the two diseases. The seat of the disease he considers to be the fibrous tissues surrounding the joints. Whilst there are points of resemblance between it and muscular rheumatism, there are also other distinctions than those of situation. There are more signs of inflammation in articular rheumatism, and its phenomena are sometimes witnessed on the external surface. It appears more frequently to originate without apparent external cause; it is more mobile; it is much oftener accompanied with fever, and, as a general rule, is of much longer duration. Articular rheumatism is generally ushered in by a number of febrile symptoms, which but rarely precede muscular rheumatism; and it is accompanied by other symptoms, varying in severity with the number of diseased articulations. When a limb is attacked with muscular rheumatism, it is rare that the joints contiguous

* Morgagni, Epit. lvii. 17.

to the rheumatic muscles become affected; but the converse is not true: and, lastly, the articular is less amenable to treatment than the muscular rheumatism. The importance attached by the author to the study of causes has given rise to some long chapters on the subject; but, in our opinion, he omits to notice the most important consideration of all,—viz. the relation of the different forms of the disease, or, as we would say, the relation of the two different diseases, gout and rheumatism, to these causes respectively. It is admitted by M. Chomel that there must be an internal *predisposition*, although we know not in what this consists. There is also a great disposition to recurrence of the disease, when once the body has been attacked by it; and the oftener that it has happened, the greater is the danger of its return: this return being occasioned frequently by the most trifling causes, but most frequently succeeding to an impression of cold, excess at table, or too much venereal indulgence. A regular and temperate mode of living is the best method of preventing relapses, which are occasionally found to be periodic. The disease is hereditary, a fact established by M. Chomel in thirty-six cases out of seventy-two; and this number he regards as sufficient to show that such a mode of transmission is certain. No predisposing causes are of such importance as the hereditary disposition and the previous occurrence of an attack. All the external predisposing causes are obscure. It is not denied that the combined action of cold and moisture contribute much to engender the rheumatic disposition. This fact is illustrated both by the history of the disease in different climates and in different seasons. Youth and manhood appear to be the ages most subject to attacks: the first attack is generally between the fifteenth and thirtieth year. Of 73 cases, M. Chomel found 35 who had been first attacked between the ages of fifteen and thirty years; 22 between thirty and forty years; 7 between forty-five and sixty; 7 after sixty years; 2 before fifteen, one aged nine, the other aged ten years. When the disease attacks young individuals, it is almost always indicative of hereditary disposition. Men are more subject to the disease than women; the sanguine than the other temperaments. The *occasional causes* of rheumatism would have required or obtained from M. Chomel little notice, had not M. Bouillaud maintained that they reduced themselves to a single one,—the action of cold, particularly in combination with moisture. The observations of the latter are all of a positive character: he is evidently too zealous an admirer of pathological laws. Of fifty patients whom he carefully examined, all owed his or her rheumatism to cold. But, since the controversy which has existed on this question, nine patients, who entered the Hôtel Dieu during November and December, 1835, and January, 1836, were most carefully questioned; and in two only of these cases did cold appear to have acted as the determining cause.* We quite agree with M. Chomel that cold is not a *constant* and *necessary* antecedent in the production of acute articular rheumatism; that, among other causes, it may be occasioned by the suppression of various long-established or habitual discharges, &c. But, in the consideration of M. Bouillaud's opinions, we must remember that he has not contended for the identity of rheumatism

* We shall hereafter refer to these nine cases, recorded by Dr. Grisolle, chef de clinique, in the Journal Hebdomadaire, No. 13. 1836.

and gout; and, unless the strict meaning of these words is laid down,—unless, as a preliminary question, it is decided whether they mean to MM. Bouillaud and Chomel the same or different diseases, it is clear that no conclusions can be satisfactorily arrived at as to the influence of cold as an exciting cause. As a cause of what is called acute articular rheumatism in this country, it is doubtless most frequent; as a cause of what we term gout, it is probably rare: and herein partly lies the difference in the opinions of our authors,—M. Bouillaud, who recognizes rheumatism as a distinct disease, attributing it to cold and wet; M. Chomel, who associates all the forms of gout with rheumatism, finding that some other cause besides cold must be sought for to explain its occurrence. M. Chomel has added two cases to those already recorded by Dr. Murray, in the Edinburgh Medico-Chirurgical Journal, of rheumatism occurring in the decline of scarlatina.

It would be superfluous to give a minute description of acute articular rheumatism; but there are some facts, and several questions arising out of them, well deserving our attention.

*Invasion.* The fever of invasion, says M. Chomel, is generally long and intense in proportion to the number of inflamed joints, and hence it is of some value as a guide in prognosis. With the partial rheumatism (i. e. of one or two joints,) there is but little fever; though during the nocturnal reaction there may be some: but, if the fever is very strong, internal organs should be most carefully examined, to ascertain whether any internal affection coincide with the rheumatism.

If no internal affection exist, there is no doubt that a general rheumatism is on the point of declaring itself. The increase and diminution of disease in a single joint do not take place in a continuous manner, but with exacerbations during the night and remissions during the day, both when the disease is on the increase and the decrease. Recurrence of disease is more frequent after partial than after general rheumatism: hence this partial rheumatism is important as a signal of attacks to come. Of itself, it is of short duration, and ceases spontaneously after fifteen days at the latest. Febrile symptoms announce acute general rheumatism; doubtless the product of a hidden cause which is about to show its special character by fixing on the joints. There is nothing in the fever which marks its speciality. The general symptoms persist sometimes for two or three days before the joints are affected. The view of the nature of the disease to which these facts lead is, that rheumatism belongs to the class of essential fevers. We shall hereafter notice M. Chomel's remarks on them. M. Bouillaud, however, is quite of the contrary opinion: he sneers at essential fevers, as mere imaginary existences; he regards this disease as a local inflammation with symptomatic fever. The general opinion, however, in this country will, we believe, be, that although the local affections may accompany the general from their first appearance, still they are not to be considered as holding the relation of cause and effect. If it be admitted (and M. Bouillaud does not allude to the fact) that the fever may precede the affection of the joints by two or three days, we cannot see on what ground the fever can be regarded as symptomatic. There are some observations on the mobility of the inflammation of the joints, made by M. Bouillaud in support of his opinion that rheumatism is an inflammation with symptomatic fever, which we here quote.

He says that this characteristic of rheumatism has not yet been investigated with sufficient care; that it is not correct to believe that acute articular rheumatism can pass from one part to another, or rather be dissipated, either easily or quickly. If, for example, the knee is much swollen, with abundant effusion into the joint, examine whether the rheumatism is terminated by *metastasis* or by *delitescence.* Two very different things are here confounded. It is true that, even in this case, the pain in the joint may suddenly disappear, with or without the occurrence of pain in another articulation; but the same cannot be said of the articular effusion, which, however, constitutes the essential element of the disease. The pain is but a symptomatic neuralgia of the affection of the joint, similar to "stitch" in pleurisy; and in each case this symptom constitutes rather an accident than an essential characteristic of the disease: for pleurisy may exist without pain, and so also may articular rheumatism, which is but, as it were, a pleurisy of the synovial membranes. To say that articular rheumatism with effusion can be suddenly displaced, is equal to supposing that pericarditis with effusion may disappear in the same manner; a hypothesis rejected by sound observation. These remarks, which we do not believe to apply to every case of acute articular rheumatism, because the effusion frequently appears to be *external* to the joint, and not within the synovial membrane, in no way confirm the assertion of rheumatism being a simple inflammation of the joint.

The paroxysms both of general and partial rheumatism, says M. Chomel, are during the night, and show themselves either by more pain or by increase of the number of joints affected. Throughout the whole course of the disease there is no necessary agreement between the fever and the articular symptoms. If the joints cease to suffer, but the fever continues, and if an examination of the viscera does not explain this by the sudden development of some internal inflammation, there is no doubt of the near reappearance of the articular pains. This is mentioned as a fact which M. Chomel was the first to point out, and a case is given, and others are referred to, in illustration. M. Bouillaud rejects as absurd the idea of a fever without an inflamed joint or organ. To explain this sort of pathological mystery, he says,—to localize *rheumatic fever without rheumatism*—it required but an attentive and exact examination of the circulating system (including the blood), and of the heart in particular. He contends that inflammation of the pericardium or endocardium is the rule in acute articular rheumatism; and that he has discovered the most satisfactory explanation of the rheumatic fever, which others have wished to *essentialize,* in the existence of a rheumatic affection of the heart, vessels, pleuræ, &c.; that these inflammations are almost always indolent, and have consequently remained undiscovered until now. In answer to the mystery supposed to attach itself to the existence of rheumatic fever, independently of local inflammation, Chomel rejoins, Does not fever precede, by twenty-four hours or more, the inflammation of the articulations? The fever is not in relation to the articular affection, as effect to cause: it is primitive, it is independent. Is it difficult to imagine the persistence of fever during the suspension of the local symptoms? There is a common cause of both, unknown but real. The phenomena of eruptive fevers and typhus are appealed to as analogous, and the case is considered as no more mysterious than those of variola, scarlatina, or measles, without the exanthema; a pathological fact now pretty generally admitted. But the question is one which must rather be decided by an appeal to facts. On the morbid anatomy of the question our authors are completely at issue, and, as it is one of considerable importance, we must enter somewhat into its details.

One object of M. Bouillaud's researches is to define the exact relation which exists between pericarditis and endocarditis, and acute articular rheumatism. Quoting from his previous work on Diseases of the Heart, he says, " Pericarditis exists in about half the number of individuals affected with acute articular rheumatism," (p. 8;) " that the pericarditis and endocarditis of rheumatism almost always accompany one another," (p. 9.) But, at page 11 of his present work, he says that, " from his calculations, it is shown that inflammation of the pericardium and endocardium coincide with articular rheumatism, in the proportion of one third." This difference is comparatively unimportant; but if, as it is maintained, the diseases can be detected during life by a skilful physician, we do not see why the proportions should be stated with so much uncertainty as to vary from one half to one third. M. Bouillaud's opinion is, that "this coincidence is the *rule*, and the non-coincidence the *exception*." The symptoms of pericarditis occurring in an individual affected with acute articular rheumatism are, " dullness of the præcordial region, much more extended than in the normal state, (twice or thrice in every direction;) an arching of the same region; pulsations of the heart remote, little or not at all sensible to the touch; sounds of the heart distant, obscure, accompanied by different abnormal sounds,—some depending on the friction of the opposite surfaces of the pericardium, others on the complication of valvular endocarditis. Pain more or less acute of the præcordial region, palpitations, irregularity, inequality, and intermittence of the pulse are sometimes associated with the preceding symptoms." With these symptoms M. Chomel is agreed, and he does not wish to throw any discredit on M. Bouillaud's cases: but he claims for his own observations the same credit, and alludes to those already mentioned, as recorded by M. Grisolle, in which, after a most careful examination, seven out of nine were found to be entirely free from any sign or symptom of heart affection. He, therefore, most properly objects to any pathological *law* being founded on a certain number of cases, observed by one individual only. In this opinion of M. Chomel we quite concur, and for other reasons than those mentioned in his book. We shall presently see what right M. Bouillaud has to the credit of having made any discoveries as to the coincidence of rheumatism and inflammations of the heart. The fact has been long known in this country; and any one who has had an opportunity of observing cases of acute rheumatism knows that the heart is *not* affected in the majority of cases. The question as to the frequency of the coincidence is one which time only can decide. The reports relating to rheumatism, lately made by Dr. R. Macleod, in the Medical Gazette, are a valuable contribution towards such a decision. In Grisolle's cases, two out of seven showed signs of affection of the heart. Of eighty-five cases related by Dr. R. Macleod, the heart is said to have been implicated in eighteen, or in rather more than one fifth.

Much of what has preceded applies also to endocarditis. Bouillaud states that it almost always accompanies pericarditis; and "I regard its existence as certain," he adds, "when the following signs exist: *Bruit de soufflet, de râpe,* or *de scie* in the præcordial region, which is dull on percussion over a surface much more considerable than in the normal state, and is also arched, though less than in pericarditis with effusion; the pulsations of the heart lift with force the præcordial region, and are frequently irregular, unequal, intermittent, accompanied sometimes by a vibrating '*frémissement.*' The pulse is hard, strong, vibrating, intermittent, like the actions of the heart." The *differential* signs of the two affections are not always very marked; so that it is sometimes difficult to say whether pericarditis or endocarditis, or the two diseases combined, exist. Such cases are those where the inflammation of the pericardium exists without much effusion, and with the formation only of false membranes. Then the pulsations of the heart are sensible to the touch, as in simple endocarditis; and the *bruit de scie* or *de soufflet,* the *frémissement* of the præcordial region, may be found as in the case of endocarditis. We have already expressed the opinion, whilst commenting on M. Bouillaud's treatise on Diseases of the Heart, "that, in the present state of our knowledge, the distinction (between endocarditis and pericarditis) will often be impossible." (*Brit. and For. Med. Review,* vol. ii. p. 328.) There is nothing in the above catalogues of symptoms to make us alter that opinion. The law (as it is termed) of the coincidence of endocarditis with acute articular rheumatism is, in M. Bouillaud's work, an inference from symptoms alone; for the cases which he treated recovered. To say the least, then, there is no foundation for the law but very uncertain symptoms. In one only of the seven cases of Grisolle was there a *bruit de soufflet;* and in this case copious bleeding had been employed. M. Chomel thinks that, in many of the cases given by M. Bouillaud, the *bruit de soufflet* and its modifications may have been the consequence of the enormous bleedings to which he subjected them, (sometimes taking as much as eight pounds.) Dr. Marshall Hall's experiments on the occurrence of this sound from loss of blood are alluded to, and a case is given in which the loss of a considerable quantity of blood, at different times, was followed by a *bruit de soufflet;* for which such evacuations afforded the most plausible explanation. But we cannot admit this explanation, unless it be shown that the anormal sounds are always subsequent to the bleedings, and that they do not coexist with other signs of affections of the heart. There is an unfairness of criticism in many of the remarks of both the authors, the avoidance of which would have obtained them credit for a desire for the prevalence of truth, instead of for that of their own favorite opinions. M. Chomel does not reject the possibility of endocarditis, but quite the contrary: he gives a case which died, and afforded an opportunity of proving the point. With regard to the precise frequency of the occurrence of endocarditis with rheumatism, we do not know where the facts are to be found on which a safe opinion can be formed. It is a question, as we have said, for time to decide, and one which those who have opportunities of examining cases of rheumatism will do well to bear in mind.

There are other serous inflammations to be looked for with acute articular rheumatism: these are chiefly in the pleura, rarely in the arachnoid or peritoneum. During the disease, internal organs, and particularly the chest, must be carefully examined. That he may not be taken by surprise, M. Chomel examines the chest every two or three days; even in the complete absence of all apparent disturbance of the circulation and respiration. This is a most valuable caution, and one which, from our own experience, we should strongly urge,—if it required to be urged by anything beyond the knowledge of the insidious and latent form which these inflammations assume.

It is remarkable that, notwithstanding what has been so long known, in this country, of the connexion of rheumatism with inflammation of the heart, and considering the great contributions made by French pathologists to our knowledge of cardiac diseases, that Corvisart only incidentally names rheumatism in conjunction with gout, as the source of one of the three forms of adhesion of the pericardium to the heart; that Laennec leaves unnoticed all such causation; whilst M. Bertin, in his excellent work on Diseases of the Heart and large Vessels, published but twelve years ago, in the composition of which M. Bouillaud was his collaborator, and M. Louis, in his treatise on Pericarditis, observe a similar silence.

The question of priority of discovery has been warmly contested by our two authors. Bouillaud says that "the subject of his researches which is most novel and curious is, without contradiction, the coincidence of inflammation of the external and internal sero-fibrous tissue of the heart with acute articular rheumatism. The date of the publication of his volume is 1836, but he states that he had collected the cases which enabled him to discover this important relationship about three years before, viz. in 1833. We have seen reason to doubt the evidence which he has given in favour of the frequency of such coincidence; and therefore, before examining at all the history of the discovery, we should repeat that, although pericarditis is found to exist frequently with acute articular rheumatism, it is not so in half or one-third of the cases; and that there is not the same amount of proof in favour of the existence of endocarditis which there is in favour of pericarditis. We think that M. Chomel has satisfactorily shown that the charge of ignorance of the occurrence of rheumatism and pericarditis, which M. Bouillaud makes against him, is unfounded; and that, in 1813, in an inaugural thesis he had written, "I have myself seen pericarditis succeed to rheumatism, and cause the death of the patients." In 1826, M. Chomel wrote in the "Dictionnaire," in twenty-one volumes, "Pericarditis has been most often observed either with pleurisy or pneumonia, *or in the course of acute articular rheumatism.*" Bearing in mind how much we consider to be proved of the coincidence of pericarditis and rheumatism, M. Chomel may fairly claim precedence of M. Bouillaud, at least in having expressed such knowledge. But M. Chomel does not claim the merit of having discovered rheumatic pericarditis. He regards it as a discovery made "par tout le monde, peu à peu par le travail commun." In fact, M. Bouillaud appears to have been singularly ignorant of the prevalent opinions on this subject, and to have discovered what almost everybody else, at all acquainted with the literature of medicine, must very well have known. In this country, at least, such has been the case. Dr.

Baillie, in his Morbid Anatomy, informs us that Dr. Pitcairn was "the first person who made this important observation;" and so long ago (as we are told by Dr. Elliottson, *Diseases of the Heart*, p. 9,) as the year 1788. Since the publication of Dr. Baillie's work in 1797, the same fact has been noticed by innumerable English writers, and, among others, by Sir David Dundas in 1809, and Dr. Wells in 1812. In 1824, it was formally noticed in Dr. Cox's compilation, On Rheumatism and its Metastasis; and still more so, in 1826, by Dr. Hawkins, in a work principally dedicated to the subject. In Dr. Elliottson's Lumleyan Lectures, read in 1829, we not only find the complication noticed as a fact generally known, but the very same practical caution given as that announced by M. Chomel. He says, "I make it as invariable a rule to examine the cardiac region by the touch and hearing, in every case of acute rheumatism, as the usual seats of hernia are examined by us all in cases of colic and intestinal inflammation." It is also but justice to Dr. Elliottson to state that he refers the affection of the heart, in all cases, to pericarditis in the first instance.

We have stated our doubts as to the extreme frequency of the complication of endocarditis with acute rheumatism, as mentioned by M. Bouillaud; and we must be allowed to tell him, also, that the fact (although he regards it as new) has been long known on this side of the Channel. In the very excellent work of Dr. Brown, of Sunderland, published ten years ago, the writer, describing the appearances found on dissecting cases of rheumatic cardiac affection, after stating that the pericardium is the principal seat of the disease, thus expresses himself: "The membrane lining the organ has participated in the affection. On detaching the abundant lymph which adheres strongly to the valves and chordæ tendineæ, it is generally found highly vascular."* It is more formally noticed by Dr. Elliottson, in the Lectures above referred to. At a period much later, but certainly preceding the publication of M. Bouillaud's work, although not anterior to his observations, the recognition of the affection was still more fully described by Dr. Watson, in two clinical lectures delivered at the Middlesex Hospital, February 28, and March 4, 1835, and which were afterwards published in the Medical Gazette. A brief extract from the first of these very excellent discourses will suffice to show Dr. Watson's opinions as to the nature and frequency of the affection termed endocarditis by M. Bouillaud.

After detailing the general symptoms of the affection of the heart, which accompanies or follows acute rheumatism, and which affection he states to be of "extreme frequency," he proceeds:

"And what are the parts upon which the inflammation fastens? They are the membranous parts of the heart; its investing membrane alone, or its lining membrane alone, or (what is infinitely the most common) both of these membranes together. On the exterior of the heart, it is probable that the inflammation begins in the fibrous texture of the pericardium, and then extends rapidly to the serous. Here it produces its usual consequences—the effusion of serum; the deposition of lymph; adhesion, general or partial, of the opposite surfaces of the membrane. The inflammation usually spreads over the greater portion, or even the whole of the serous surface; &c." . . . "When the inner membrane is affected (and very seldom, I believe, does it escape, although its alterations may sometimes elude an inattentive observer), the

* Medical Essays, by J. Brown, M.D. p. 159.—*London*, 1828.

inflammation exercises (if I may so speak) a kind of preference; its effects are limited, in a great measure, to the valvular apparatus of the heart. Occasionally that naturally transparent portion of the membrane which covers the muscular fibres is thickened, and rendered whitish and opaque; and occasionally some of the deposits that are common on the valves encroach also somewhat beyond them, or even stud, here and there, the interior of the several cavities. But the valves, or the cartilaginous rings from which they spring, are the parts first and chiefly implicated, especially the mitral valve, and the aortic valves, not uncommonly the tricuspid valve also; and sometimes even the semilunar valves of the pulmonary artery." . . . "The valves themselves become thicker—they lose their transparency and pliancy—they become puckered; sometimes they are folded down and glued, as it were, to the opposite surface; but more frequently than all, they present small wart-like granulations or excrescences—what the French call *vegetations*." . . . "These are the appearances commonly seen when the patient does not long survive the first attack of rheumatic carditis; for under that name I may now speak of the disease we are considering. When death takes place at a later period, you find more than this; you find the consequences which flow from these primary lesions operating as mechanical causes of further change—hypertrophy, and dilatation, in their various degrees and combinations."

In his zeal against M. Bouillaud's opinions, M. Chomel states that the pleura is as often inflamed in acute rheumatism as the pericardium. This is but a statement, and one which we believe is incorrect. Andral, however, gives, in his Clinique Mèdicale, two cases, (vol. ii. p. 502-504;) one of which was latent, the other not so. Affections of the peritoneum and arachnoid membrane in acute rheumatism are rare.

Except in the cases mentioned, says M. Chomel, the *termination* of acute articular rheumatism is favorable, and rheumatic pericarditis and pleurisy are less dangerous than the ordinary forms of these diseases. Can it end in suppuration? Cases have been recorded, apparently of such termination. But, since pathological anatomy has been more studied, it is very probable that metastatic abscesses, and purulent collections after common inflammation have been set down as instances of rheumatism ending in suppuration. Chomel says that pathological anatomy has been as vainly interrogated with regard to acute articular as with regard to muscular rheumatism; and that, to speak truly, there is none, either for the one or the other, in the actual state of science. Bouillaud is, of course, of the contrary opinion; and instances cases, in his book, of rheumatism ending in suppuration. To some of these, only, has Chomel answered; and as, in these cases, there was phlebitis, or other conditions than those of simple rheumatism, we agree with him in his objections to them. At page 85, however, of M. Bouillaud's book, is a case, communicated by M. Raciborzki, which appears to be a fair example of rheumatism ending in suppuration. There is certainly a great want of evidence on this subject; but, while we doubt the frequency of suppuration as an effect of acute articular inflammation, we by no means agree with M. Chomel that it has never been shown to exist. Few as the cases are, says M. Bouillaud, they serve to show that the termination by suppuration or purulent effusion is not foreign to acute articular rheumatism, and that is all that could be claimed for it.

Our two authors are at issue, also, as to the *duration* of acute articular rheumatism. M. Bouillaud says that it is in relation to the treatment: M. Chomel thinks that treatment cannot with any certainty stop or even abridge it. He says that its duration is very uncertain; that its average is three weeks; that this uncertainty renders any inferences as to the action of medicines very doubtful. What are called *good* cases show themselves under the most different forms of treatment; i. e. cases which last from twelve to sixteen days. This spontaneous termination is, of course, a very important matter to decide, in estimating the value of any treatment. We believe that Louis has lately suffered several cases of acute rheumatism to take their own course, and that he has found their average duration to be three weeks. Show us a medicine, says M. Chomel, which, in forty cases, always cures in fourteen days, and then there will be no reason to doubt its efficacy. However, although we cannot dissent from M. Chomel's principle, we should not conclude that a great degree of probability was not afforded of the utility of any system of treatment, if, *cæteris paribus*, it considerably reduced the average duration of the disease. We shall pass over the squabbles of the rival doctors on this question; the object of M. Bouillaud being to show that hitherto the duration of the disease has been very long, that he may magnify the more his own system of treatment; and of M. Chomel to reduce this average duration as much as possible. If they were not such useful physicians, we should almost lament that they had not been educated as advocates, they are such thorough partisans. Having got the clue, you can tell at once what their conclusions will be, on many points; a circumstance very unsatisfactory for readers who cannot participate in their insignificant animosities.

Chomel examines successively the various *remedies* which have been employed in cases of acute rheumatism. *General Bleeding.* His own practice is, neither to bleed profusely nor to interdict bleeding; employing it as a means simply of moderating the fever. We need not follow him in his examination of the various authorities for or against bleeding. M. Bouillaud's opinion on the subject is thus expressed: "The true specific of acute articular rheumatism, its *quinine*, if the expression is allowable, is the antiphlogistic system, and bleeding is the prince of antiphlogistics." But this *quinine* of rheumatism is bleeding after a regulated formula, termed "*coup sur coup.*" It does not merely concern the quantity of blood taken, but the mode in which it is taken. Its panygeric shall precede the description of this mode.

"The success obtained by this new formula of bleeding is such, that unless it was seen, it could not actually be credited. I am not therefore surprised at the *philosophic scepticism* of some individuals. . . . By the use of the new formula, the average duration of acute rheumatism, is, from one to two weeks, only, instead of from six to eight." [The formula is as follows.] "*The day* on which the patient enters (supposing him of strong constitution and at a vigorous age) at the evening visit, he is bled to sixteen ounces. (In very sanguineous subjects, this bleeding is sometimes carried to twenty or twenty-four ounces.) *Second day.* Two bleedings of from fourteen to sixteen ounces, and in the interval of the two bleedings, a local bleeding either by leeches or cupping (the latter is preferred). By this local bleeding, twelve, sixteen, or twenty ounces of blood are drawn. The cupping-glasses are applied around the most inflamed articulations, and upon the præcordial region, when the heart is seriously affected, that is to say, in the great majority of cases. *Third day.* A fourth bleeding similar to that of the evening of the previous day, and a second cupping (from twelve to sixteen ounces) either upon the precordial region or around the articulations. *Fourth day.* The fever, the pains, the swelling, in a word, the whole inflammation sometimes ceases from the fourth day. In this case, further bleeding is not performed; in the contrary case, another bleeding of twelve or sixteen ounces is performed. *Fifth day.* In general, the resolution of the disease is in full activity. But in very serious cases, the rheumatic fever may still be very marked; and twelve ounces of blood are drawn from the arm, or the same quantity is taken locally. From the sixth, seventh, or eighth day, the convalescence is established, and nourishment may be commenced. If serious relapses occur (the *new formula* does not secure the patient against them, but perhaps exposes him to them less than the *old*) it may be necessary to recur to the bleeding. It is thus that in a case where four bleedings had stopped (jugulé) a serious attack of acute rheumatism, a violent relapse took place, and this could only be subdued by five new bleedings. If the relapses are mild, emollients, abstinence, baths, opiates, &c. may suffice. To prevent relapses, the patient should most carefully guard against any exposure to cold. The additional means to bleedings thus practised, are,—abstinence, emollient drinks, blisters, compression of the diseased articulations, the application of mercurial ointment upon compresses; position; emollient poultices; baths; opium at a moderate dose, internally or endermically. The medium quantity of blood taken from vigorous subjects, in a violent case, is from four to five pounds. In some cases it may be necessary to draw six, seven, or even eight pounds of blood. But in moderate cases the dose drawn need not be more than two or three pounds." (p. 133.)

Such is the plan of bleeding, "*coup sur coup*," of M. Bouillaud. Of course, the only claim to any novelty in this system must be the manner in which it is performed; and those who have instanced the inutility of large bleedings in their hands receive as a reply from M. Bouillaud, "you did not bleed according to my formula;" and we must admit the fairness of the reply; although the objection would hardly apply to the practice prevalent in Edinburgh some thirty or forty years since, while the Cullenian doctrines and practice were still in full force. When we remember that acute rheumatism is not a fatal disease; that it runs a certain course when no treatment is employed; that, under the influence of remedies well known in this country, and of which, in the practice of many, bleeding never forms a part, its termination is favorable; we are utterly unable to justify such sanguinary practice as this of M. Bouillaud. Were it shown that it was a means of materially abridging the disease, we should regard this alone as a very trifling argument in its favour. We ourselves would rather suffer a certain number of days from rheumatism than from the after-consequences of the loss of four, five, six, seven, eight pounds of blood! Of the history of these patients after they have left the hospital we hear nothing, but it is the most important part of it. The cure is considered complete when the poor wretch has left the hospital free from rheumatic pains; the hospital which receives him next is left to the reader's imagination. But, without denying any of M. Bouillaud's facts, M. Chomel has taken some pains to show that the treatment does not abridge the average duration of the disease. We regard the average duration as not very well settled, and some exception might be taken to M. Chomel's observations. Facts already stated (and, among others, the spontaneous termination of the disease,) will excuse our entering into the niceties of M. Chomel's calculations to show that this new treatment cannot, on the evidence produced, be said to shorten the duration of the complaint. In the main we agree with M. Chomel, although he has taken some little advantage of his opponent in some points of his calculations.

In his remarks on the effects of *sudorifics* in the treatment of acute rheumatism, M. Chomel does not speak from experience. Theoretically he condemns them; but we must protest against all theoretical condemnation of any remedy which is spoken well of by many who have employed it; and, although the cases are few in which, from our own experience, we should regard sudorifics as appropriate, we know that they are highly estimated by others. In the Infirmary of Edinburgh they have been long employed; and this would argue somewhat in favour of their utility. Narcotics generally, but especially opium, are commended when the fever has lost its primitive intensity, or if the disease was originally apyretic. But on this point M. Chomel says nothing satisfactory. Of the use of the medicine he knows evidently very little or nothing. In fact, his whole chapter on Treatment is by far the worst in the book. Like many others of his class in Paris, the history, diagnosis, pathology of a disease, with hot squabbles about unimportant matters, absorb all his talents and zeal; and his feebleness shows itself in the discussion of its treatment. The use of opium in acute rheumatism is a matter of considerable importance; and there can be no doubt that it occasionally acts in a way justly entitling it to the name of *heroic*. This result we have ourselves witnessed in some cases; and we think that, if preceded by due depletion, it is often not merely a safe but a very valuable remedy. The attention of the profession in this country was particularly called to this remedy by Dr. De Roches, in a paper published in the first volume of the Ed. Med. and Surg. Journal; although it had been often employed before, in different forms. Sydenham was adverse to the use of opium in this disease, but in his opinions respecting its proper treatment, he appears to have been much influenced by theoretical considerations. Dr. Heberden, in speaking of rheumatism, remarks, "Meo judicio, opium non tantum modo importuni mali præsidium est, sed multum confert ad ipsum malum tollendum." In the paper of Dr. De Roches referred to above, the author states that the induction of perspiration is essential to the success of the remedy, and our own experience confirms this: unless perspiration is excited, the patient's sufferings will be aggravated, in place of being relieved. The fulfilling this indication seemed the chief object of treatment at the Edinburgh school in the beginning of the present century. The late Dr. Gregory recommended, with this view, the administration of Dover's powder in doses of ten grains every two or three hours, with warm diluent drinks and close wrapping in blankets. We are far from recommending the indiscriminate employment of opium, in any form, in acute articular rheumatism, but we are convinced that it as little merits to be rejected as entirely useless.

The moderate use of *purgatives* is not objected to by M. Chomel; but the preference is given to clysters, particularly as they can be administered without requiring that the patient should be disturbed. Indeed, one of the chief objections to the use of purgatives in the disease is, that any movement of the body is attended with so much pain. As usual, M. Chomel misunderstands and misrepresents the views with which calomel is employed in this country in acute rheumatism. He appears to have no idea of its actions, except as a purgative and a sialogogue, and he consequently speaks ignorantly of "cette panacée de la tourbe medicale de l'empire Brittanique." We cordially agree with him in condemning its still too frequent employment; but he must have witnessed the indubitable effects of this mineral, when neither employed to purge nor to salivate, before he can be considered as a competent judge in the matter.

*C[illegible]chicum* he used in one case, on which he thus comments: "in short, colchicum appears to us to be a remedy little to be relied on." This very short condemnation is an example of the nature of much of his remarks on the treatment of rheumatism. If, theoretically, he disapproves of a remedy, or if in one case he has found it fail, or if he has not attended to the conditions under which its employment (by those in the habit of using it) is recommended, he does not hesitate to exclude it as a remedy unworthy of consideration. His disapproval does not, in the case of colchicum, appear to be at all influenced by the fact that "it is used with very happy effects in the large hospital of Westminster" (a remarkable instance, by the bye, of his acquaintance with what is going on in this country,) or that his "savant confrère et ami le docteur Magliari" has published instances of "de remarquables succès avec le vin de colchìque." It is not necessary for us to point out to our readers the great value of this remedy in rheumatism, and its proper mode of employment, as these are generally well understood and appreciated in this country. For the best information which we know on the subject,—and we may add for much important information of a pathological kind not to be found in the works of MM. Chomel and Bouillaud,—we refer our younger readers to the two admirable articles, On Gout and Rheumatism, by Dr. Barlow, in the *Cyclopædia of Practical Medicine*.

Of *Quinine* and *Digitalis* M. Chomel has had no experience. *Tartar emetic* he has employed, without having any reason to repeat it. A solitary case to which *mercurial frictions* were applied was an instance of failure; "so that we are authorized in considering mercurial frictions as entirely deficient in efficacy in cases of acute articular rheumatism." This most legitimate inference would almost lead us to suspect that these frictions had been recommended by M. Bouillaud. In acute, partial, or apyretic articular rheumatism, *the endermic application of the salts of morphium* is recommended, on the experience of two cases which are quoted.

The *principles of the rational treatment* (as it is called) of rheumatism are, *according to M. Chomel*, established "conformably to an eclecticism counselled by experience;" although it is not easy to gather from the remarks made by him on the remedies above mentioned, whether this eclecticism means the same to him that it does to other people. We should rather think not. His system appears to us to exclude eclecticism; to be founded on his own views of the nature of the disease; a treatment, in fact, based on hypothesis. Be this as it may, the rational treatment of this disease, according to M. Chomel, is as follows:—Bleeding, in young and robust subjects, once or twice, to moderate the fever and the local inflammations, and to prevent metastasis; but this should never be carried to such a degree as to diminish the strength considerably; for weakness lengthens convalescence and predisposes singularly to relapse. Local is less useful than general bleeding; but, if the pain is so intolerable in any joint that there are convulsive movements of adjoining muscles, it must be employed. Leeches are better than cupping, because their application is attended with much less pain. Sometimes emollient poultices are useful. Warm baths, though indicated, give rise, from the lifting of the patient, to great pain. Cooling drinks, such as whey, &c., are pleasant. The apartment should be of moderate temperature; 60° Fahr. The bed should be firm rather than soft; never a feather-bed. When the pains are very acute, and are much increased by motion, a bed should be used where the patient is lifted by means of straps. Position is important, and should be such as to allow the free reflux of blood from the inflamed parts. The duration of the disease is such that a too rigorous abstinence must not be enjoined. Towards its termination, diaphoretics are advantageous: the best of these are vapour baths. The occurrence of pericarditis or pleurisy during the course of the disease, requires, of course, to be vigorously treated. As far as these rules of treatment extend, they quite meet our approval, although M. Chomel does not give us cases illustrative of its advantages. When he adds to his remedies colchicum, and employs it judiciously; when he is aware of the real object with which calomel is employed in this country; and, we may add, when he has discriminated the cases in which sudorifics are apparently useful, we doubt not that he will rewrite his chapters on treatment, and thereby very much increase their utility; and that he will treat his patients with more success, and perhaps be disposed to think that the disease is capable of being abridged by the adoption of an " eclecticism counselled by experience."

*Chronic articular rheumatism* has so great an analogy with the acute that we shall chiefly deal with its points of difference. It has two very distinct forms; the one succeeding to the acute, the other commencing with its own proper characteristics. Each of these may be subdivided into the *mild* and *intense.*

*Mild Chronic Articular Rheumatism.* This differs from the mild acute form only in its minor degree of mobility, and in its duration. Instead of an excess of heat, it produces often a constant sensation of cold in the part. The variations in its course are the same as those of the *intense chronic articular rheumatism.* Here there is complete inability to move the joint, which is generally swollen and deformed,—the joint surrounded by hard and prominent tophaceous concretions. If these concretions are recent, they may be gradually dissipated; if of long standing, this is no longer possible. They gradually render the skin which covers them thinner: they are chiefly observed in the smaller articulations. If limited to a few articulations, the disease does not endanger life: if, however, very intense, attacking many articulations, and giving rise to violent fever, death may follow. Sometimes eschars form, subsequent to an erythema, on those parts on which the body rests; an incurable ulcer follows, the cause of its production effecting its extension, or death takes place from suppuration. Rheumatic hectic fever sometimes extinguishes life. This termination is rare in private practice, but is frequent in hospitals for old men. In chronic articular rheumatism, there are often nocturnal exacerbations; easily calmed, however, by opiates. A damp cold season is most unfavorable; a warm dry atmosphere produces marked relief, if not a radical cure. The duration of chronic rheumatism is from three to four months; but it may be prolonged indefinitely, particularly when tophaceous concretions have taken place.

The *Prognosis* is favorable, and complete recovery may be predicted if, notwithstanding the fixedness of the pain, the articulations are still capable of being moved.

The *Diagnosis* is not always very easy. The diseases termed *white swellings*, and syphilitic diseases of the joints, are those with which rheumatic affections may be confounded. Respecting the latter, M. Chomel says that, in the syphilitic and rheumatic, several articulations may be simultaneously diseased; that, although syphilitic swellings are generally developed in the bodies, and not in the extremities of the long bones, such is not universally the case. Sometimes syphilis attacks the ends of the long bones: this is a rare but an actual occurrence. Hence the importance of diagnosis in such a case. No doubt, the articulation may, as in rheumatism, be painful, swollen, red, and heated; but commonly these symptoms are not present in the entire joint, but are limited to a part of it. Thus, for instance, they attack the acromion, one condyle of the elbow, a single styloid apophysis of the wrist, one condyle of the femur, one malleolus, &c.: but, if the syphilitic affection of the articular extremity gives rise to effusion, then there will be swelling of the entire articulation; but still, in this case, the pain is felt in a particular spot. Again, with the same degree of pain, the rheumatic patient cannot move the diseased limb: whilst, on the other hand, the syphilitic preserves all his power of motion, the pain being little if at all increased by motion. The attendant circumstances, of course, must always be borne in mind. In cases where the constitution has been saturated with mercury, M. Chomel especially recommends "*la tisane de Feltz*," which is indubitably an excellent antiphlogistic for individuals saturated with mercury. It is supposed that the active principles of this ptisan is arsenic in very small quantity, combined with sulphuret of antimony.

*Pathological Anatomy.* If the articulation has not become deformed, there is generally no appreciable lesion. Sometimes, however, curious lesions are found to exist, such as the following:—The synovial membrane was detached, and covered with small holes, the diameter of which varied from a line to a line and a half. In the points corresponding to these holes, the compact tissue of the bone was entirely destroyed; the spongy tissue alone remaining and this was of a reddish colour, but not however softened, as in caries; the medullary cavity of the bone contained a bloody serum. Sometimes, instead of articular cartilages, a cellulo-vascular tissue is found; instead of the normal layer which covers the extremities of the bones, there exist fleshy granulations, which are detached from the bony substance. Occasionally there are no traces at all of cartilaginous tissue. A superficial ulceration, or destruction, of the articular cartilage is also found, without the development of cellular vascular vegetations; the bone is almost or quite naked: this denudation does not generally exist over the whole extent of the articular extremity, but is constituted by small irregular perforations, from a line to a line and a half in diameter. M. Chomel regards this lesion as consecutive to that previously mentioned. In addition to the lesions of the synovial membranes, cartilages, and extremities of the bone, seen in M. Chomel's cases, there was an infiltration of blood into the fibro-cellular tissues external to the synovial membrane, the internal parietes of the articular cavity were of a blackish colour, depending on this hyperæmia; and, at the knee and hip, the interarticular ligaments presented the same appearance. These lesions appear to be of an inflammatory nature, and seem also to belong to chronic rheumatism, but, since they are not constantly connected with this affection, they cannot be regarded as its anatomical cause: they are probably but an effect of it. Blood has been found effused within the articulation, as well as externally to it. Suppuration has never been regarded as an effect of chronic articular rheumatism. The tophaceous concretions are seen only in a comparatively few cases, and appear to require, as a condition of their formation, some special idiosyncrasy, independent of the arthritic or gouty diathesis. These concretions are found in the most various parts of the articulations, from their interior to the layers of the skin; and sometimes, immediately beneath the epidermis, where they appear on the point of escaping. Is it hence to be inferred that this morbid secretion takes place indifferently in all the regions of the joint? Or do they not (formed originally in the fibrous tissues,) irritate and give rise to ulceration of the adjacent tissues; and hence pass, in one direction, into the articulation, in another, towards the skin? It is certain that when, either by incision or ulceration, the tophaceous matter is evacuated, the articulation is not thus necessarily opened and exposed to the external air. Hence it appears that it is not formed within the joint; but that, when it is found there, it has penetrated in the same manner, as it makes its passage through the skin: and this may perhaps be regarded as additional evidence in favour of this disease having its primitive and idiopathic seat in the fibrous tissues. From the various analyses of the secreted matter, it appears to consist chiefly of uric acid, either free or in combination with some base, such as soda or lime.

There is nothing which need detain us on the treatment of chronic articular rheumatism.

Order III. Visceral Rheumatism. This section is perhaps the most unsatisfactory in the volume, requiring, as it does, a considerable exercise of faith on the part of the reader. All the instances, recorded by authors, of gout being transported to the brain, &c., are not considered as belonging to this class of rheumatic affections. M. Chomel only recognizes internal rheumatism where there exists a muscular or fibrous tissue, such as is found in, 1, the diaphragm; 2, the heart; 3, the air-tubes, which present, in the larynx, a complete system of articulations; 4, the alimentary canal, (pharynx, œsophagus, stomach, and intestines); 5, the bladder; 6, the uterus: and, before further considering the rheumatic affections of these various parts, he says, "that in no particular case can the disease be admitted or diagnosticated except on presumptive evidence, not by an absolute demonstration;" although he considers that their existence may be contested "*en thèse générale.*"

1. *Rheumatism of the Diaphragm.* This may be suspected in individuals subject to or actually affected by regular rheumatism, if there be a sensation of painful constriction at the base of the thorax with dyspnœa. The physician called to such a case will be especially struck with the increase of pain at the attachments of the diaphragm, on inspiration, on the passage of food or drink, or on eructation of wind. With these symptoms may be connected cough and hiccough. This last symptom is regarded as important and characteristic. Sauvages speaks of *Singultus arthriticus* as a symptom of metastasis of gouty matter to the diaphragm.

The treatment recommended for this, as well as for other visceral rheumatisms is, to recall the rheumatism into parts the functions of which can be disturbed with less inconvenience; such as the articulations, and particularly those previously affected. In cases of danger, the revulsions must be of a powerful and quickly operating kind; such as boiling water and ammoniacal ointment; continuing at the same time more tardy counter-irritation, by mustard, &c.

2. *Rheumatism of the Heart.* M. Chomel thinks that this may be either acute or chronic. Many diseases of the heart, regarded as incurable organic affections, but which have perfectly recovered, it may be suspected, were rheumatic. The muscularity of the heart would lead us to suspect that it might become rheumatic. The above remark on the cures which have taken place of affections of the heart, regarded as organic and incurable, is, we believe, especially appropriate to the young; and it is one which deserves more consideration than our author has given to it. It must have occurred to every one to meet with children, either actually rheumatic or born of rheumatic parents, with signs and symptoms of affection of the heart, such as have led those generally considered good judges of diseases of that organ, to condemn them as incurable, which children have however subsequently lost every symptom of cardiac affection: and these cases are not such as admit of explanation by supposing the occurrence of pericarditis, or indeed of any action leading to organic change. Syncope of short duration, occurring during the course of acute articular rheumatism, a palpitation, more or less considerable, happening under similar circumstances, and disappearing at the end of two or three hours, leaving no traces behind it; in short, some anomalous symptoms referrible to the heart, gone almost as soon as they appear, indicate very probably acute cardiac rheumatism. But its more common form is the chronic. Some gouty or rheumatic people are subject to præcordial pains, not only whilst walking quickly or going up stairs, but suddenly and during the night. There may be commencing anasarca, in consequence of disturbed circulation, and all these formidable symptoms may cease as by enchantment, on the occasion of a regular attack of rheumatism or gout.

These are the symptoms which M. Chomel ascribes to the two forms of cardiac rheumatism. Its differential diagnosis requires great care; and, as examination after death cannot confirm it, it is the more essential to pay great attention to the symptoms existing during life. From *Pleurodynia* it may be distinguished, because, in this disease, the pain is increased by pressure and by inspiration. *Pericarditis* has its dull sound on percussion, excepting at first; and there is fever. *Organic diseases of the heart* have their dependent signs. *Nervous palpitations* occur from a thousand physical or moral causes, in nervous individuals. There has here been no previous rheumatism; and most commonly not the slightest increase of sensibility in the region of the heart precedes the sudden invasion of palpitations. *Angina pectoris* is a disease very analogous to this rheumatism, and it is supposed that it may sometimes be rheumatism. If the above symptoms, evidently attached to the heart, cannot be ascribed to any of the previous diseases, and they occur in a rheumatic subject or in one whose parents were rheumatic, there is some probability of the existence of cardiac rheumatism. The same treatment is required as in the former case.

Under the head of *Rheumatism of the Air-Tubes*, M. Chomel merely alludes to instances of aphonia occurring in the course of rheumatism, which may be of a rheumatic character; the disease existing in the larynx.

*Rheumatism of the Alimentary Canal.* The ancients have spoken of this disease in the stomach and intestines. It has of late been more disregarded, since it has been more the practice to seek for anatomical characteristics of disease. At the beginning of this century, Barthez published some excellent observations on acute and chronic gout of the stomach and intestines; and Rodamel has published facts tending to establish the reality of rheumatic pains in the stomach. M. Chomel believes that gastro-intestinal neuralgia is somewhat rare; and that the affections generally termed gastralgia and enteralgia, in opposition to gastritis and enteritis, should rather be regarded as metastasis of rheumatism, or of affections of the skin (*dartres*); that, in the former case it is the muscular coat, in the latter the mucous, which is the seat of disease. He contends that the structure of the stomach and intestines afford *à priori* evidence of this doctrine, and that it is also confirmed by facts. The *chronic form* is that most frequently observed. If the stomach is rheumatic, the epigastric sensibility is increased, and indeed much more than it is commonly in inflammation of its mucous membrane; and this pain, which affords a singular contrast with the absence or feeble degree of febrile movement, is especially increased by pressure. Besides this, there is nausea and even vomiting; but the vomited matters are not such as are proper to inflammation. A similar pain in the part affected marks rheumatism of the intestines, and there are irregular colic pains. In each case the pain may vary a hundred times in intensity and situation, without, on the whole, being influenced by food. This is contrary to what happens in inflammation: in rheumatism, there is nothing uniform in the effects produced by ingestion of food, nor in the character of the alvine evacuations. Sometimes the pain is increased, at others diminished, whilst sometimes it remains wholly unchanged. It is not uncommon to observe this in many who are said to be affected with chronic gastritis or enteritis. Sometimes such people are better after eating more than usual, or after taking exciting food. This want of influence of food proves that there exists some other affection than inflammation. It is particularly after certain atmospheric conditions that the pains become more acute, and especially when the air is cold and moist, or is stormy and charged with electricity. Patients complain that they have been bled in vain, condemned to abstinence, obliged to submit to a vigorous antiphlogistic regimen, without becoming either better or worse. They have found temporary effects only from calmatives. Previously, most of them have had either articular or muscular rheumatism, or may have it still, or are born of rheumatic or gouty parents. By the aid of these considerations, gastric and intestinal rheumatism may be recognized; confounded, as they have been, by M. Broussais with inflammations, with neuralgia by M. Pinel. Apyretic and frequently transient pains, or pains which last an indefinite period without general *malaise*, are the best means by which the physician may recognize, whether in the acute or chronic forms, a rheumatic affection of the bowels. The prognosis is unfavorable as it regards the duration of the disease and the probability of its relapse. Anatomical evidence of it is sought in vain. The treatment is the same as that already mentioned; but with greater attention to food, which should not be of an exciting character.

We have given M. Chomel's remarks on this subject fully; but, as in the former chapters on visceral rheumatism, they are not quite satisfactory; although they may explain many cases, now termed gastralgia and enteralgia with much probability; and indeed, in this country, symptoms of such a character are ascribed to intestinal rheumatism. But it is probable that M. Chomel may have included cases of a different character, under the same arrangement. The absence of pain in many instances on taking food would make us hesitate whether such cases should be regarded as rheumatic; for the slightest action of a rheumatic muscle is attended with great increase of pain. Distension and certain spontaneous movements of the stomach and intestines are the consequence of taking in food. But we are here required to believe that their muscular tunics may be rheumatic, and yet that they may move without pain. In the absence of anatomical evidence to the contrary, and seeing beside no single objection to such a belief, we therefore think that many of these affections, i. e. "apyretic, and, frequently, transient pains, or pains which last an indefinite period without general malaise," are really neuralgic, whilst others are of a rheumatic character. M. Chomel is too much disposed to be exclusive in his arrangements, and we regard this as an example.

*Rheumatism of the Bladder.* The acute form of this succeeds another rheumatism suddenly interrupted, or it occurs in the course of acute articular rheumatism. Sometimes, there is frequent micturition, and the excretion is very painful; sometimes there is retention. It would thus seem that the disease was at one time in the neck, at another in the fundus of the bladder. Occasionally, in the same individual, there are observed, in the same attack, alterations of dysuria and ischuria. It may also, says M. Chomel, exist in the chronic form. The diagnosis in these cases is to us quite unsatisfactory.

There is nothing that need delay us in the remarks on *Rheumatism of the Uterus.* Rheumatism is believed by M. Chomel to exist *in the Peritosteum,* in those cases where there are fixed pains on a superficial bone, without any trace of swelling, and which appear consecutively to, or simultaneously with, affections which are incontestibly rheumatic. So likewise, some pains in *the teeth* are regarded as *rheumatic;* an instance of which is given in which shifting pains from the upper to the lower jaw succeeded to an attack of lumbago and articular pains. It is conjectured that the *dura mater* may be affected with rheumatism. M. Chomel restricts the application of the term *arthritic inflammation of the sclerotic* more so than is generally done. He recognizes it, when the sclerotic becomes the seat of pain, and the redness of the conjunctiva is consecutive without catarrhal secretion, and when these things take place in an individual previously affected with rheumatism, with which this affection has alternated.

We have already stated that M. Chomel regards gout and articular rheumatism as the same disease, and he now examines the grounds on which the distinction, admitted by others, rests. We will here abridge his opinions. Is the distinction made, he remarks, because gout affects the small joints, and particularly those of the great toe, to the exclusion of other joints? It is evident that the symptoms of articular rheumatism should not be exactly the same at the hip and at the phalanges. In rheumatism of a large joint, there is little or no evident swelling; in that of one of intermediate size, the redness and swelling are more marked; still more evident are they when a small joint is affected. But these, instead of constituting essential differences, are but dependent on the greater or less quantity of tissues interposed between the joint and the skin. Besides, in many *gouty* individuals, both large and small joints are simultaneously attacked: and if, as is indicated by the use of the generic name, a striking analogy is allowed to exist between muscular and articular rheumatism, there is greater reason to admit a relationship between what is termed gout and articular rheumatism; for, between the two last, the resemblance is much more striking than between articular and muscular rheumatism. M. Chomel has found that the digestive organs are not particularly troubled in what is termed gout, more than in ordinary and simple inflammation of the great joints: he has rather noticed the reverse. Rheumatism is hereditary, so that this quality cannot be regarded as peculiarly belonging to gout; nor can its rapid change of seat or the periodicity of its attacks. All the forms under which gout is described apply exactly to articular rheumatism. The *regular gout* is in no way different from it. The *atonic gout* is simply a very natural complication of muscular rheumatism with the gastro-intestinal rheumatism already described; indeed this should rather be termed muscular rheumatism than gout; for in this case neither large nor small articulations are affected: and, in *metastatic gout*, when there is the sudden explosion of inflammation in some viscus, consequent on the delitescence of the articular affection, what is this more than the metastasis which so frequently happens in rheumatism, and which is no less frequent when the large than when the small joints are attacked? There appears, then, to be nothing, as far as it concerns the *seat* of the disease, which separates gout or inflammation of the small articulations, as essentially different from rheumatic inflammation of the large joints. It is said that the poor and labouring classes are spared, and that the rich suffer from the gout. A poor man does not come to an hospital on account of a rheumatic finger or toe; he does not seek medical aid until he is forced to do so, by having lost, more or less, the use of his limbs. But it would be a mistake to suppose that amongst the poor the disease never commences in the small joints. Of this M. Chomel has seen two instances, and some have likewise occurred to M. Requin, the editor of M. Chomel's work, in his dispensary practice. The tophaceous concretions, too, cannot be regarded as signs of gout as a disease distinct from rheumatism; for, if a person is simultaneously affected with the disease in the small and large joints, it is only in the former that these concretions take place. This must be attributed only to a difference in the situation, and not in the nature of the disease: otherwise, how can it be explained that the gout (so called) where it passes from the toes to the knee, so rarely occasions the formation of concretions in the latter? It must therefore be admitted, concludes M. Chomel, that there is no legitimate and fundamental distinction between gout and common articular rheumatism: gout, consequently, in good nosology, should be regarded as identical with rheumatism, or, at the most, as a simple variety of it.

We have given M. Chomel's reasons without thinking that they at all decide the question. In the present state of our knowledge of the diseases termed gout and rheumatism, we are probably not justified in absolutely maintaining their identity or non-identity. As good a case might be made out in favour of the identity of some of the exanthemata, as is here attempted for gout and rheumatism. It is unfortunate that in this instance, as well as in other diseases, we can consider but a few of their phenomena; others, as the state of the fluids, &c., escaping our notice; and that our decision of the question is to be made on this partial knowledge. Hence, the arguments drawn by M. Chomel from what he terms identity in the *seat* of the disease, are uncertain and inconclusive. It is from this circumstance, probably, that M. Chomel's observations, although pointing out more marks of similarity than have hitherto been noticed between gout and rheumatism, are to us quite unsatisfactory. We are still disposed to regard these diseases as distinct, although in many parts, both local and constitutional, strikingly allied. Some of the arguments adduced by M. Chomel will not be admitted as founded on facts; and there are others to which he has not alluded, to which, in approaching to a decision of the question at issue, some weight may probably be attached. Let the reader bear in mind well marked cases of regular gout (as it is termed) and acute articular rheumatism; and we think that he will have no hesitation in recognizing in the former, as a general rule, a state of absolute or relative plethora which is not found in the latter; and, although we are ready to admit, with M. Chomel, that actual dyspepsia is fully more prevalent in rheumatic than in gouty individuals, still we cannot doubt that temporary disorder of a digestion generally healthy, is vastly more frequent as a precursor of gout than of rheumatism. Few experienced practitioners in this country will admit that the poor are as obnoxious to gout as the rich; or, rather, the penurious livers, as the luxurious: and we all know that the facilities of obtaining gratuitous medical assistance in this country would never prevent a poor man's applying for it, if he were suffering from what is termed an attack of gout; a disease quite as disabling for a time as any attack of acute articular rheumatism. There is likewise a distinction in the degree and kind of pain; "sometimes resembling the tension or laceration of the ligaments, sometimes the gnawing of a dog," as Sydenham describes it, from his personal experience. The causes are likewise regarded as in some measure distinct; those of gout being internal, whilst rheumatism more often arises from exposure to cold and wet. The ages at which they occur, notwithstanding the calculations of M. Chomel, (and these calculations, it must be remembered, are made assuming the identity of the diseases,) we still regard as in some degree marking the peculiarity of the diseases. The effect of treatment is likewise somewhat different: colchicum in gout being almost specific; not so in rheumatism. But, notwithstanding all that can be said in favour of the distinctness of the two diseases, they appear very nearly to resemble one another, and to run together in those cases which are termed *rheumatic gout*: and these are the cases which afford the strongest support to M. Chomel's opinions. The general sense of the profession is in favour of the relationship of gout and rheumatism, and it is more accordant with nature to suppose that one runs into the other by insensible gradations, than to believe that there exists any well-defined boundary line between them. Man makes these divisions for his own convenience; they are not the work of nature. Between the animal and vegetable there are intermediate structures, which cannot be referred exclusively to one or the other kingdom; and between the natural families of plants and animals there is the same gradual transition. So it is with diseases; and, in this instance, is not rheumatic gout the union of two diseases which must be regarded as distinct? Can it be the case that, from their different mode of life, or from some other cause, gout is a rare disease among the French, and that consequently M. Chomel is not very conversant with it? He has identified the rheumatism and gout, and considered both under one name; but, certainly, whilst as a treatise on what we term rheumatism, we should highly recommend his book, we should regard it as trustworthyless as a treatise on gout; feeling assured that any practical and experienced physician, who has been in the habit of attending the wealthier classes in England and witnessing their gout, can have no difficulty in drawing the distinction between such cases and the rheumatism of the lower classes.*

M. Chomel terminates his work by some remarks on the nature of rheumatism, regarding it as a disease *sui generis*, which requires to be classed by itself in a nosological arrangement. We will give his reasons shortly. In many cases, rheumatism appears with all the phenomena of inflammation; but it has been seen that this is not always the case. Pain is sometimes the only symptom of rheumatism: to this may be added heat, or, on the contrary, a sensation of cold. The pain, also, is frequently not increased by pressure, but is relieved by rubbing. In muscular rheumatism, no change of structure can be detected after death; and that form of the disease which is most inflammatory is distinguished from simple phlegmasia. The inflammation is not limited to one point, but scattered over several; and, as often as we find on the surface of or within the body many spots *(foyers)* of inflammation,—as, for example, in variola, &c.—our reason is obliged to admit a unique and common cause of all these disseminated inflammations; and it is this cause (call it virus, miasm, or what you will,) which constitutes the essence of the disease, and which should never be lost sight of for the sake of partial inflammations, which are indeed its most frequent manifestation, but which may in fact be absent; the disease remaining essentially the same. So variola may exist without pustulation; typhus fever without inflammation of Peyer's glands: and thus the cause of rheumatism does not always affect the joints, but may disturb, immediately, idiopathically, and independently, certain viscera. The disappearance of rheumatism from a joint, and its sudden appearance elsewhere, is quite unlike a simple phlegmasia. Its want of fixed periods, of determinate duration, distinguish it also from simple inflammation; and, during the course of rheumatic fever, the joints may be freed from disease whilst the fever continues. Hence it follows that inflammation does not constitute the whole of the disease. There is no recorded example of the disease ending in gangrene, and its termination in suppuration is not an established fact. Hence it follows that rheumatism is not to be arranged among phlegmasiæ, properly so called; and that, when it does present itself in an inflammatory form, the inflammation is not idiopathic but symptomatic, and that it has a specific nature. M. Bouillaud's opinion is expressed only on acute articular rheumatism; in favour of the inflammatory character of which he advances the usual arguments. With respect to the multifarious forms of disease included by M. Chomel under

* We would once more refer to Dr. Barlow's article on *Gout*, in the Cyclopædia, for the best diagnosis we know of gout and rheumatism. Vol. ii. p. 364.

the term rheumatism, many doubtful questions might be raised; and the first is, whether there has been more than presumptive evidence to show that what is called muscular is the same disease as articular rheumatism? We have not room at present to moot the question, nor to apply all the arguments which have been over and over again used by the opponents and advocates of idiopathic fevers, and diseases caused by a morbid poison, to this subject of rheumatism. We want more knowledge before such a discussion could be brought to anything like a satisfactory conclusion. At the same time, common sense has long ago convinced us of the inflammatory nature of acute rheumatism, although we qualify it by terming it a specific inflammation; and hence it is not to be judged of according to the rules of common inflammation: the disease must be studied by itself.

For such a study we can recommend both the works before us. M. Chomel's will give many a reader a clearer notion of rheumatism than he previously possessed, although he may think that the term has been allowed too extensive an application. Like all the productions of eminent men of the French school, it is remarkably methodical and free from all obscurity: its fault, which equally belongs to the same school, is too great diffuseness, too much explanation, too little belief in the reader's own powers of reflection and judgment. The fondness for their own exclusive views, also, is an objection to both the works before us: but to the readers of the present article, this, together with the other defects, is of less importance, as the analysis presents most of the valuable matter, and all which can be regarded as novel, contained in the two books; together with (it is hoped) an impartial criticism of those subjects concerning which the authors' opinions are particularly at variance, or where their importance, or novelty, or any other quality, appeared to render them worthy of notice.

---

### Art. VII.

*The Transactions of the Provincial Medical and Surgical Association.* Vol. VI.—*London and Worcester,* 1838. 8vo. pp. 621.

